# On 3-Coloring of ($2P_4,C_5$)-Free Graphs

**DOI:** 10.1007/s00453-022-00937-9

**Published:** 2022-02-15

**Authors:** Vít Jelínek, Tereza Klimošová, Tomáš Masařík, Jana Novotná, Aneta Pokorná

**Affiliations:** 1 Faculty of Mathematics and Physics, Charles University, Malostranské Náměstí 25, 11800 Prague, Czech Republic; 2 Faculty of Mathematics, Informatics and Mechanics, University of Warsaw, Banacha 2, 02-097 Warsaw, Poland; 3 Department of Mathematics, Simon Fraser University, 8888 University Drive, Burnaby, BC V5A 1S6 Canada

**Keywords:** 3-Coloring, Hereditary classes, $2P_4$-Free graphs, Cographs, 05C75

## Abstract

The 3-coloring of hereditary graph classes has been a deeply-researched problem in the last decade. A hereditary graph class is characterized by a (possibly infinite) list of minimal forbidden induced subgraphs $H_1,H_2,\ldots$; the graphs in the class are called $(H_1,H_2,\ldots )$-free. The complexity of 3-coloring is far from being understood, even for classes defined by a few small forbidden induced subgraphs. For *H*-free graphs, the complexity is settled for any *H* on up to seven vertices. There are only two unsolved cases on eight vertices, namely $2P_4$ and $P_8$. For $P_8$-free graphs, some partial results are known, but to the best of our knowledge, $2P_4$-free graphs have not been explored yet. In this paper, we show that the 3-coloring problem is polynomial-time solvable on $(2P_4,C_5)$-free graphs.

## Introduction

Graph coloring is a notoriously known and well-studied concept in both graph theory and theoretical computer science. A *k*-*coloring* of a graph $G=(V,E)$ is defined as a mapping $c:V\rightarrow \{1,\ldots ,k\}$ which is *proper*, i.e., it assigns distinct colors to $u,v\in V$ if $uv\in E$. The *k* -coloring problem asks whether a given graph admits a *k*-coloring. For any $k\ge 3$, the *k*-coloring is a well-known NP-complete problem [2]. We also define a more general *list-k-coloring* where each vertex *v* has a list *P*(*v*) of allowed colors such that $P(v)\subseteq \{1,\ldots ,k\}$. In that case, the coloring function *c*, in addition to being proper, has to respect the lists, that is, $c(v)\in P(v)$ for every vertex *v*.

A graph class is *hereditary* if it is closed under vertex deletion. It follows that a graph class ${\mathcal {G}}$ is hereditary if and only if ${\mathcal {G}}$ can be characterized by a unique (not necessarily finite) set ${\mathcal {H}}_{{\mathcal {G}}}$ of minimal forbidden induced subgraphs. Special attention was given to hereditary graph classes where ${\mathcal {H}}_{{\mathcal {G}}}$ contains only one or only a very few elements. In such cases, when $\{H\}={\mathcal {H}}_{{\mathcal {G}}}$, or $\{H_1,H_2,\ldots \}=\mathcal H_{{\mathcal {G}}}$, we say that $G\in {\mathcal {G}}$ is *H*-*free*, or $(H_1,H_2,\ldots )$-*free*, respectively. We let $P_t$ denote the path on *t* vertices, and $C_\ell$ the cycle on $\ell$ vertices. We let ${\overline{H}}$ denote the complement of a graph *H*. For two graphs $H_1$ and $H_2$, we let $H_1+H_2$ denote their disjoint union, and we write *kH* for the disjoint union of *k* copies of a graph *H*.

In recent years, a lot of attention has been paid to determining the complexity of *k*-coloring of *H*-free graphs. Classical results imply that for every $k\ge 3$, *k*-coloring of *H*-free graphs is NP-complete if *H* contains a cycle [3] or an induced claw [4, 5]. Hence, it remains to consider the cases where *H* is a *linear forest*, i.e., a disjoint union of paths. The situation around complexity of (list) *k*-coloring on $P_t$-free graphs where $k\ge 4$ has been resolved completely. The cases $k=4,t\ge 7$ and $k\ge 5,t\ge 6$ are NP-complete [6] while cases for $k\ge 1,t=5$ are polynomial-time solvable [7]. In fact, *k*-coloring is polynomial-time solvable on $sP_1+P_5$-free graphs for any $s\ge 0$ [8]. The borderline case where $k=4,t=6$ has been settled recently. There the 4-coloring problem (even the precoloring extension problem with 4 colors) is polynomial-time solvable [9] while the list 4-coloring problem is NP-complete [10]. Hajebi et al. show that 5-coloring on $2P_4$-free graphs is NP-complete [11].

Now, we move our focus towards the complexity of the 3-coloring problem, which was less well understood, in spite of the amount of research interest it received in the past years. However, a considerable progress has been made in 2020; a quasi-polynomial algorithm running in time $n^{O(\log ^2(n))}$ on *n*-vertex $P_t$-free graphs (*t* is a constant) was shown by Pilipczuk et al. [12], extending a breakthrough of Gartland and Lokshtanov [13]. In the realms of polynomiality, Bonomo et al. [14] found a polynomial-time algorithm for $P_7$-free graphs. Klimošová et al. [15] completed the classification of 3-coloring of *H*-free graphs, for any *H* on up to 7 vertices. These results were subsequently extended to $P_6+rP_3$-free graphs, for any $r\ge 0$ [16]. There are only two remaining graphs on at most 8 vertices, namely $P_8$ and $2P_4$, for which the complexity of 3-coloring is still unknown.

Algorithms for subclasses of $P_t$-free graphs, which avoid one or more additional induced subgraphs, usually cycles, have been studied. They might be a first step in the attempt to settle the case of $P_t$-free graphs. This turned out to be the case for 3-coloring of $P_7$-free graphs (as can be seen from preprints [17–19] leading to [14]) and 4-coloring of $P_6$-free graphs [20].

Note that the problem of 4-coloring is NP-complete even when some $(P_t,C_\ell )$-free graphs are considered when $t\ge 7$. Hell and Huang [21] and Huang et al. [22] settled many NP-complete cases of this type. These results, in combination with the polynomiality of $P_6$-free case, leave open only the following cases: $(P_7,C_7)$-free, $(P_8,C_7)$-free, and $(P_t,C_3)$-free graphs, for $7\le t\le 21$.

Chudnovsky and Stacho [23] studied the problem of 3-coloring of $P_8$-free graphs which additionally avoid induced cycles of two distinct lengths; specifically, they consider graphs that are $(P_8,C_3,C_4)$-free, $(P_8,C_3,C_5)$-free, and $(P_8,C_4,C_5)$-free. For the first two cases, they show that all such graphs are 3-colorable. For the last one, they provide a complete list of 3-*critical graphs*, i.e., the graphs with no 3-coloring such that all their proper induced subgraphs are 3-colorable. Independently, using a computer search, Goedgebeur and Schaudt [24] showed that there are only finitely many 3-critical ($P_8, C_4$)-free graphs. In fact, 3-coloring is polynomial-time solvable on ($P_t,C_4$)-free graphs for any $t\ge 1$ [25].

The situation concerning $2P_4$ or $P_8$ is still far from being determined when two forbidden induced subgraphs are considered; in particular, it is not known whether $(P_8,C_3)$-free, $(P_8,C_5)$-free, $(2P_4,C_3)$-free, or $(2P_4,C_5)$-free graphs can be 3-colored in polynomial time.^1^ This is in contrast with the algorithm for $(P_7,C_3)$-free graphs [28] which is considerably simpler than the one for $P_7$-free graphs [14]. Recently, Rojas and Stein [27] approached the problem by showing that for any odd $t\ge 9$, there exists a polynomial-time algorithm that solves the 3-coloring problem in $P_t$-free graphs of odd girth at least $t-2$. In particular, their result implies that 3-coloring is polynomial-time solvable for $(P_9,C_3,C_5)$-free graphs.

Freshly, a similar question was resolved in the case where, instead of a cycle, a 1-subdivision of $K_{1,s}$ (a star with *s* leaves), denoted as $SDK_{1,s}$, is considered. Chudnovsky, Spirkl, and Zhong have shown that the class of $(SDK_{1,s},P_t)$-free graphs is list-3-colorable in polynomial time for any $s,t\ge 1$ [29]. For other related results and history of the problem, please consult a recent survey [26].

In this paper, we resolve one of the remaining open problems mentioned above, which considers $2P_4$-free graphs, as we will describe a polynomial-time algorithm for 3-coloring of $(2P_4,C_5)$-free graphs. To the best of our knowledge, this is a first attempt to attack the 3-coloring of $2P_4$-free graphs.

### Theorem 1

The 3-coloring problem is polynomial-time solvable on $(2P_4,C_5)$-free graphs.

To prove our result, we will make use of some relatively standard techniques. Let $\omega (G)$ be the size of the largest clique of graph *G*. We use a seminal result of Grötschel et al. [30] that shows the *k*-coloring problem on *perfect graphs*, i.e., graphs where each induced subgraph $G'$ is $\omega (G')$-colorable, can be solved in polynomial time. Perfect graphs are characterized by the strong perfect graph theorem [31] as the graphs that have neither odd-length induced cycles nor complement of odd-length induced cycles on at least five vertices.

As $K_4$ and $\overline{C_7}$ graphs are not 3-colorable, we can assume that our graph is $(2P_4,C_5,\overline{C_7},K_4)$-free. As $K_4\subseteq \overline{C_\ell }$ whenever $\ell \ge 8$ and $2P_4\subseteq C_\ell$ whenever $\ell \ge 10$, it follows that either the graph is perfect, or it contains $C_7$ or $C_9$. In the first case, we are done by the aforementioned polynomial-time algorithm. For the latter cases, we divide the analysis into two further subcases. First, we suppose that the graph is $(2P_4,C_5, C_7,\overline{C_7},K_4)$-free and therefore it must contain $C_9$. We analyze this case in Sect. 2.1. Second, we suppose that graph contains $C_7$ and we analyze this case in Sect. 2.3.

We will exploit the fact that once we find an induced $P_4$, the vertices that are not adjacent to it must induce a $P_4$-free graph (also known as *cograph*). Such graphs were among the first *H*-free graphs studied and have many nice properties, e.g., any greedy coloring gives a proper coloring using the least number of colors [32]. We will make use of a slightly stronger statement that handles the list-3-coloring problem.

### Theorem 2

[26] The list-3-coloring problem on $P_4$-free graphs can be solved in polynomial time.

The 3-coloring algorithm that we develop to prove Theorem 1 cannot be directly extended to solve the more general list-3-coloring problem since it uses the 3-coloring algorithm for perfect graphs to deal with graphs avoiding $C_7$ and $C_9$. However, apart from this one case, the algorithm works with the more general setting of list-3-coloring. In fact, we use reductions of lists as one of our base techniques. After several branching steps with polynomially many branches and suitable structural reductions of the original graph *G*, the algorithm will transform a 3-coloring instance of a $(2P_4,C_5)$-free graph *G* to a set of polynomially many heavily structured list-3-coloring instances. These structured instances can then be encoded by a 2-SAT formula, whose satisfiability is solvable in linear time [33].

## Proof of Theorem 1

We are given a $(2P_4,C_5)$-free graph $G=(V,E)$, and our goal is to determine whether it is 3-colorable. We will present an algorithm that solves this problem in polynomial time. The algorithm begins by checking that the graph is $\overline{C_7}$-free, and that the neighborhood of each vertex induces a bipartite graph, rejecting the instance if the check fails. Note that this check ensures, in particular, that *G* is $K_4$-free.

The algorithm then partitions the graph into connected components, solving the 3-coloring problem for each component separately. From now on, we assume that the graph $G=(V,E)$ is connected, $\overline{C_7}$-free, and each of its vertices has a bipartite neighborhood.

The basic idea of the algorithm is to choose an initial subgraph $N_0$ of bounded size, try all possible proper 3-colorings of $N_0$, and analyze how the precoloring of $N_0$ affects the possible colorings of the remaining vertices.

We let $N_1$ denote the vertices in $V{\setminus } N_0$ which are adjacent to at least one vertex of $N_0$, and we let $N_2$ be the set $V{\setminus } (N_0\cup N_1)$. We will use the notation *N*(*x*) for the set of neighbors of *x* in *G*, and $N_i(x)$ for $N_i\cap N(x)$.

Our algorithm will iteratively color the vertices of *G*. We will assume that throughout the algorithm, each vertex *v* has a list $P(v)\subseteq \{1,2,3\}$ of *available colors*. We call *P*(*v*) the *palette of v*. The goal is then to find a proper coloring of *G* in which each vertex is colored by one of its available colors. The problem of deciding the existence of such coloring is known as the *list-3-coloring problem*, and is a generalization of the 3-coloring problem.

Whenever a vertex *x* of *G* is colored by a color *c* in the course of the algorithm, we immediately remove *c* from the palette of *x*’s neighbors. Additionally, if the vertex *x* is not in $N_0$, it is then deleted. The vertices in $N_0$ are kept in *G* even after they are colored. We then update the list-3-coloring instance using the following *basic reductions*:

- If a vertex *y* has only one color $c'$ left in *P*(*y*), we color it by the color $c'$ and remove $c'$ from the palettes of its neighbors. If $y\not \in N_0$, we then delete *y*.
- If *P*(*y*) is empty for a vertex *y*, the instance of list-3-coloring is rejected.
- If, for a vertex $y\not \in N_0$, the size of *P*(*y*) is greater than the degree of *y*, we delete *y*.
- *Diamond consistency rule*: If *y* and $y'$ are a pair of nonadjacent vertices such that $P(y)\ne P(y')$, and if $N(y)\cap N(y')$ is not an independent set, then any valid 3-coloring of *G* must assign the same color to *y* and $y'$; we therefore replace both *P*(*y*) and $P(y')$ with $P(y)\cap P(y')$.
- *Neighborhood domination rule*: If *y* and $y'$ are a pair of nonadjacent vertices such that $N(y)\subseteq N(y')$ and $P(y')\subseteq P(y)$, and if *y* is not in $N_0$, we delete *y*.
- If *G* has a connected component in which every vertex has at most two available colors, we determine whether the component is colorable by reducing the problem to an instance of 2-SAT. If the component can be colored, we remove it from *G* and continue. Otherwise, we reject the whole instance.
- If a connected component of *G* is $P_4$-free, we solve the list-3-coloring problem for this component by Theorem 2. If the component is colorable, we remove it. Otherwise, we reject the whole instance *G*.

It is clear that the rules are correct in the sense that the instance of list-3-coloring produced by a basic reduction is list-3-colorable if and only if the original instance was list-3-colorable. It is also clear that we may determine in polynomial time whether an instance of list-3-coloring (with fixed $N_0$) permits an application of a basic reduction, and perform the basic reduction, if available. Throughout the algorithm, we apply the basic reductions greedily as long as possible until we reach a situation where none of them is applicable.

The basic reductions by themselves are not sufficient to solve the 3-coloring problem for *G*. Our algorithm will sometimes also need to perform branching, i.e., explore several alternative ways to color a vertex or a set of vertices. Formally, this means that the algorithm reduces a given instance *G* of list-3-coloring to an equivalent set of instances $\{G_1,\dotsc ,G_k\}$; here saying that a list-3-coloring instance *G* is *equivalent* to a set $\{G_1,\dotsc ,G_k\}$ of instances means that *G* has a solution if and only if at least one of $G_1,\dotsc ,G_k$ has a solution.

At the beginning of the algorithm, we attach to each vertex *v* of *G* the list $P(v)=\{1,2,3\}$ of available colors, thereby formally transforming it to an instance of list-3-coloring. The algorithm will then try all possible proper 3-colorings of $N_0$, and for each such coloring, apply basic reductions as long as any basic reduction is applicable. If this fails to color all the vertices, more complicated reduction steps and further branching will be performed, to be described later.

Overall, the algorithm will ensure that the initial instance *G* is eventually reduced to a set of at most polynomially many smaller instances, each of which can be transformed to an equivalent instance of 2-SAT, which then can be solved efficiently.

### The $C_7$-Free Case

Our choice of $N_0$ will depend on the structure of *G*. More precisely, if *G* contains an induced copy of $C_7$, we will choose one such copy as $N_0$. This is by far the most challenging case, and we return to it later.

The case when *G* is $C_7$-free can be handled in a simple way, as we now show.

#### Proposition 3

The 3-coloring problem for a $(2P_4,C_5,C_7)$-free graph *G* can be solved in polynomial time.

#### Proof

Recall that we assume that *G* is $K_4$-free and $\overline{C_7}$-free; otherwise *G* would clearly not be 3-colorable. Note that $K_4$-freeness implies that *G* is $\overline{C_k}$-free for every $k\ge 8$, and $2P_4$-freeness implies that *G* is $C_k$-free for every $k\ge 10$.

If *G* is also $C_9$-free, then it is perfect by the strong perfect graph theorem, and since it is $K_4$-free, it is 3-colorable. Assume then that *G* contains an induced copy of $C_9$. Fix $N_0$ to be an induced copy of $C_9$ in *G*, and define $N_1$ and $N_2$ accordingly. We will show that for any proper coloring of $N_0$, the basic reductions can solve the resulting list-3-coloring problem.

Fix a 3-coloring of $N_0$, and apply the basic reductions until none of them is applicable. We claim that this solves the instance completely, i.e., we either color the whole graph, or determine that no coloring exists. For contradiction, suppose that we reached a situation when *G* still contains uncolored vertices, but no basic reduction is applicable.

It follows that *G* contains a vertex *v* with three available colors, and this vertex necessarily belongs to $N_2$. As $N_2$ is also $P_4$-free the connected component containing *v* is not an isolated component within $N_2$. Therefore, we may find in *G* two adjacent vertices *x*, *y* with $x\in N_1$ and $y\in N_2$. Recall that $N_0(x)$ is the set of vertices of $N_0$ adjacent to *x*. The vertices of $N_0(x)$ partition the cycle $N_0$ into edge-disjoint arcs, and at least one of these arcs has an odd number of edges. Let *A* be such an arc of odd length.

**Fig. 1:**
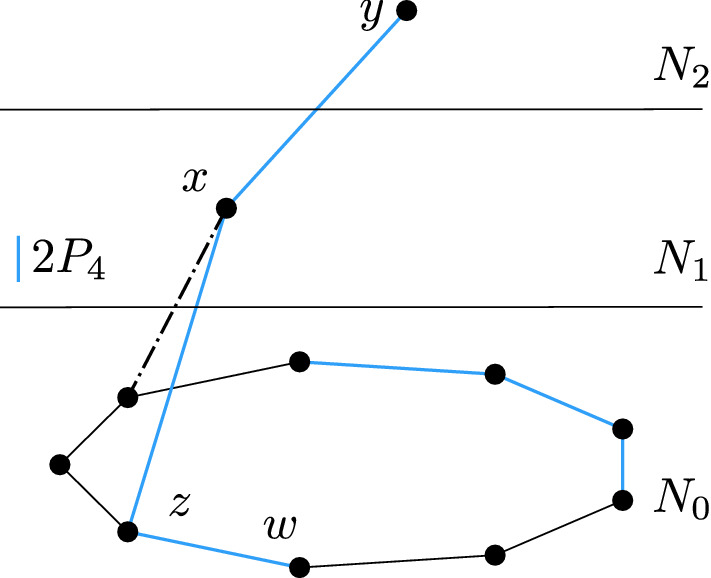
Picture showing the induced $2P_4$ in the case of *G* being $C_7$-free. If the dash-and-dotted edge is present, *A* has length 7, otherwise *A* has length 9

If *A* has length 1, then *x* is adjacent to two adjacent vertices of $N_0$, hence the color of *x* is uniquely determined by the coloring of $N_0$ and *x* should have been deleted. If *A* has length 3 or 5, then $A\cup \{x\}$ induces a copy of $C_5$ or $C_7$, respectively, which is impossible. Thus, *A* has length 7 or 9. In such case, we find a copy of $2P_4$ in *G*, where one $P_4$ consists of *y*, *x*, a vertex $z\in N_0(x)$, and a vertex $w\in A$ adjacent to *z*, while the other $P_4$ is formed by taking four consecutive internal vertices of *A*, each of which is at distance at least two from *z* and *w*; see Fig. 1. In all cases we get a contradiction. $\square$

From now on, we assume that the graph *G* contains an induced $C_7$. We choose one such $C_7$ as $N_0$, and define $N_1$ and $N_2$ accordingly.

### More Complicated Reductions

We apply the basic reductions described previously whenever an opportunity arises. Now, we outline more complicated reductions which will be applied only in specific situations.

#### Cut Reduction

Suppose $G=(V,E)$ is a connected instance of list-3-coloring. Let $X\subseteq V$ be a vertex cut of *G*, let *C* be a union of one or more connected components of $G-X$, and let $C_X$ be the subgraph of *G* induced by $C\cup X$. Suppose further that the following conditions hold.

- *C* has at least two vertices.
- *X* is an independent set in *G*.
- All the vertices in *X* have the same palette, which has size 2.
- For any two vertices $x,x'$ in *X*, we have $N(x)\cap C= N(x')\cap C$.
- The graph $C_X$ is $P_4$-free.

Assume without loss of generality that all the vertices of *X* have the palette equal to $\{1,2\}$. Let us say that a coloring $c:X\rightarrow \{1,2\}$ of *X* is *feasible for C*, if it can be extended into a proper 3-coloring of the list-3-coloring instance $C_X$. Note that the feasibility of a given coloring can be determined in polynomial time by Theorem 2, because $C_X$ is a cograph.

We distinguish three types of possible colorings of *X*: the *all-1* coloring colors all the vertices of *X* by the color 1, the *all-2* coloring colors all the vertices of *X* by color 2, and a *mixed* coloring is a coloring that uses both available colors on *X*. Observe that if *X* admits at least one mixed coloring feasible for *C*, then every (not necessarily mixed) coloring of *X* by colors 1 and 2 is feasible for *C*. This is because when we extend a mixed coloring of *X* to a coloring of $C_X$, all the vertices $y \in C$ must receive the color 3. If such a coloring of *C* exists, we can combine it with any coloring of *X* by colors 1 and 2.

The *cut reduction* of *X* and *C* is an operation that reduces *G* to a smaller, equivalent list-3-coloring instance, determined as follows. We choose an arbitrary mixed coloring *c* of *X*, and check whether it is feasible for *C*. If it is feasible, we reduce the instance *G* to $G-C$, leaving the palettes of the remaining vertices unchanged. The new instance is equivalent to the original one since any proper list-3-coloring of $G-C$ can be extended to a coloring of *G* because all the colorings of *X* are feasible for *C*.

If the mixed coloring *c* is not feasible for *C*, we know that no mixed coloring is feasible. We then test the all-1 and the all-2 coloring for feasibility. If both are feasible, we reduce the instance *G* by replacing *C* with a single new vertex *v*, with palette $P(v)=\{1,2\}$, and connecting *v* to all the vertices of *X*. Note that the reduced instance is an induced subgraph of the original one. It is easy to see that the reduced instance is equivalent to the original one.

If only one coloring of *X* is feasible for *C*, we delete *C*, color the vertices of *X* using the unique feasible coloring, and delete the corresponding color from the palettes of the neighbors of *X* in $G-C$. If no coloring of *X* is feasible for *C*, we declare that *G* is not list-3-colorable.

#### Neighborhood Collapse

Let *G* be an instance of list-3-coloring, and let *v* be a vertex of *G*. Suppose that *N*(*v*) induces in *G* a connected bipartite graph with nonempty partite classes *X* and *Y*. Suppose furthermore that all the vertices of *X* have the same palette $P_X$, and all the vertices in *Y* have the same palette $P_Y$. The *neighborhood collapse* of the vertex *v* is the operation that replaces *X* and *Y* by a pair of new vertices *x* and *y*, adjacent to each other and to *v*, with the property that any vertex of $G-Y$ adjacent to at least one vertex in *X* will be made adjacent to *x*, and similarly every vertex adjacent to *Y* in $G-X$ will be adjacent to *y*. We then set $P(x)=P_X$ and $P(y)=P_Y$. Informally speaking, we have collapsed the vertices in *X* to a single vertex *x*, and similarly for *Y* and *y*.

It is clear that the collapsed instance is equivalent to the original one. However, since the new instance is not necessarily an induced subgraph of the original one, it might happen, e.g., that a collapse performed in a $C_5$-free graph will introduce a copy of $C_5$ in the collapsed instance. In our algorithm, we will only perform collapses at a stage when $C_5$-freeness is no longer needed.

On the other hand, $2P_4$-freeness is preserved by collapses, as we now show.

##### Lemma 4

Let *G* be a $2P_4$-free instance of list-3-coloring in which a neighborhood collapse of a vertex *v* may be performed, and let $G^*$ be the graph obtained by the collapse. Then $G^*$ is $2P_4$-free.

##### Proof

Suppose $G^*$ contains an induced $2P_4$, and let *P* and *Q* be the two nonadjacent copies of $P_4$. Let *x* and *y* be the two vertices obtained by collapsing sets *X* and *Y*, as in the definition of neighborhood collapse. Without loss of generality, *P* contains the vertex *x*. It follows that *Q* contains none of *x*, *y* or *v*, and in particular, *Q* is also a $P_4$ in *G*.

If the path *P* contains the edge *xy*, we may ‘lift’ *P* into the graph *G* by replacing the vertices *x* and *y* by appropriate vertices $x'\in X$ and $y'\in Y$, and by replacing the edge *xy* by a shortest path from $x'$ to $y'$ in *N*(*v*). This transforms *P* into an induced path $P'$ in *G* on at least four vertices which is nonadjacent to *Q*. Thus, *G* also contains a $2P_4$.

Suppose now that *P* does not contain the edge *xy*, and therefore *y* is not in *P*. If *x* is the end-vertex of *P*, say $P=xw_1w_2w_3$, we easily obtain a $2P_4$ in *G* by simply replacing *x* by a vertex $x'\in X$ adjacent to $w_1$ in *G*. Suppose then that *x* is an internal vertex of *P*, say $P=w_1x w_2w_3$. Since we know that *P* does not contain *y*, we may replace the vertex $w_1$ with *v* in *P*, knowing that $vxw_2w_3$ is also an induced $P_4$ in $G^*$ nonadjacent to *Q*. By replacing the vertex *x* by a vertex $x'\in X$ adjacent to $w_2$, we obtain the induced path $vx'w_2w_3$ in *G* which forms a $2P_4$ together with *Q*. $\square$

### Graphs Containing $C_7$

We now turn to the most complicated part of our coloring algorithm, which solves the 3-coloring problem for a $(2P_4,C_5)$-free graph *G* that contains an induced $C_7$. We let $N_0$ be an induced copy of $C_7$ in this graph, and define $N_1$ and $N_2$ accordingly.

We let $v_1,v_2,\dotsc ,v_7$ denote the vertices of $N_0$, in the order in which they appear on the cycle $N_0$. We evaluate their indices modulo 7, so that, e.g., $v_8=v_1$.

Fix a proper coloring of $N_0$, and apply the basic reductions to *G* until no basic reduction is applicable. We now analyze the structure of *G* at this stage of the algorithm. We again let $N_0(x)$ denote the set of neighbors of *x* in $N_0$.

#### Lemma 5

After fixing the coloring of $N_0$ and applying all available basic reductions, the graph *G* has the following properties.

- Each vertex *x* of $N_1$ satisfies either $N_0(x)=\{v_i\}$ for some *i*, or $N_0(x)=\{v_i,v_{i+2}\}$ for some *i*.
- Each induced copy of $P_4$ in *G* has at most two vertices in $N_2$.
- *G* is connected.

#### Proof

To prove the first part, use the vertices of $N_0(x)$ to partition the cycle $N_0$ into edge-disjoint arcs. Note that none of these arcs has length 1. Since then, *x* would be adjacent to two vertices of distinct colors, and it would have been colored and deleted. Also, none of these arcs can have length 3, since such an arc together with the vertex *x* would induce a $C_5$ in *G*, contradicting $C_5$-freeness.

On the other hand, at least one of the arcs formed by $N_0(x)$ must have an odd length. Thus, there is either an arc of length 7, implying $N_0(x)=\{v_i\}$, or there is an arc of length 5, implying $N_0(x)=\{v_i,v_{i+2}\}$ for some *i*. This proves the first part of the lemma.

**Fig. 2:**
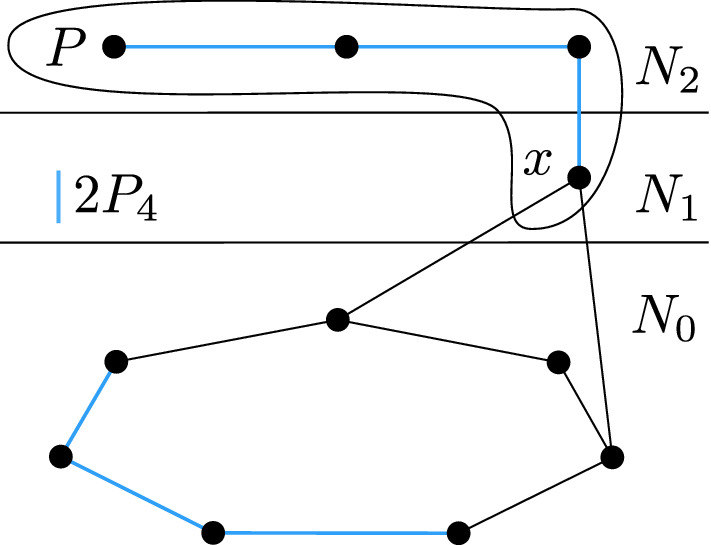
Finding an induced $2P_4$, assuming *P* is an induced $P_4$ with exactly three vertices in $N_2$. Note that *P* can look differently, but always contains *x*

To prove the second part, assume that *P* is an induced copy of $P_4$ in *G* with at least three vertices in $N_2$. If *P* is fully contained in $N_2$, then *P* forms a $2P_4$ together with any $P_4$ contained in $N_0$. Suppose that $P{\setminus } N_2$ consists of a single vertex *x*, as in Fig. 2. Necessarily *x* is in $N_1$, and by the first part of the lemma, $N_0{\setminus } N_0(x)$ contains an induced $P_4$ which forms an induced $2P_4$ with *P*.

To prove the last part of the lemma, note that $N_0$ is connected and therefore contained in a single component of *G*, and if *G* contained another connected component, then this other component would necessarily be $P_4$-free and would be colored by a basic reduction. $\square$

Lemma 5 is the last part of the proof that makes use of the $C_5$-freeness of *G*. From now on, we will not need to use the fact that *G* is $C_5$-free. In particular, we will allow ourselves reduction operations, such as the neighborhood collapse, which do not preserve $C_5$-freeness.

We will assume, without mentioning explicitly, that after performing any modification of the list-3-coloring instance *G*, we always apply basic reductions until no more basic reductions are available.

In the rest of the proof, we use the term *top component* to refer to a connected component of $N_2$. Observe that every top component is $P_4$-free and therefore has a dominating set of size at most 2 [34]. We say that a top component is *relevant*, if it contains a vertex *z* with $|P(z)|=3$. Note that if *G* has no relevant top component, then all its vertices have at most two available colors, and the coloring problem can be solved by a single basic reduction.

We will say that a vertex *x* of $N_1$ is *relevant* if *x* is adjacent to a vertex belonging to a relevant top component.

Let $x\in N_1$ be a vertex, and let *C* be a top component. We say that *x* is a *partial neighbor* of *C*, if *x* is adjacent to at least one but not all the vertices of *C*. We say that *x* is a *full neighbor* of *C*, if it is adjacent to every vertex of *C*.

#### Lemma 6

Suppose $x\in N_1$ is a partial neighbor of a top component *C*. Then *x* is not a neighbor of any other top component. Moreover, $|N_0(x)|=2$.

#### Proof

**Fig. 3:**
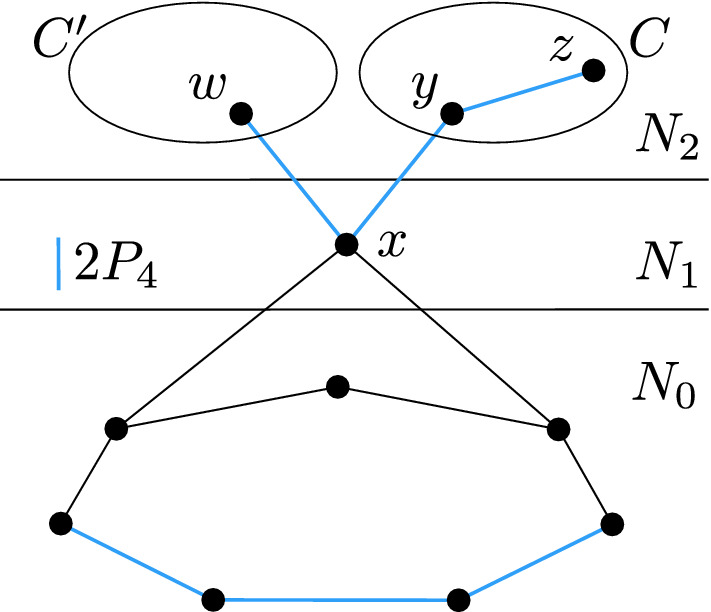
Vertex $x \in N_1$ being a partial neighbor of a top component *C* and neighboring another top component $C'$ leads to an induced $2P_4$

Let *y* and *z* be two adjacent vertices belonging to *C*, such that *x* is adjacent to *y* but not to *z*. Suppose for contradiction that there is a vertex $w\in N_2$ adjacent to *x* but not belonging to *C*. Then *wxyz* is a copy of $P_4$ with three vertices in $N_{2}$, as shown in Fig. 3, which contradicts Lemma 5. This shows that *x* is not adjacent to any top component other than *C*.

Suppose now that $N_0(x)$ contains a single vertex $v_i$. Then $v_ixyz$ together with $v_{i+2}v_{i+3}v_{i+4}v_{i+5}$ induce a $2P_4$. $\square$

We will now reduce *G* to a set of polynomially many instances in which the set of relevant vertices has a special form. We first eliminate the relevant vertices that have only one neighbor in $N_0$. Let $R_i$ be the set of relevant vertices that are adjacent to $v_i$ and not adjacent to any other vertex of $N_0$.

**Fig. 4:**
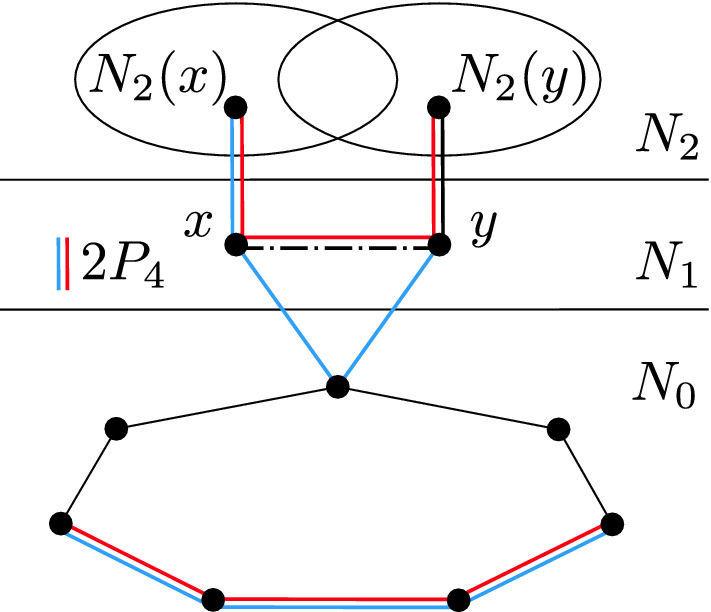
The situation obtained from the assumption that $N_2(x)$ and $N_2(y)$ for $x,y \in R_i$ are not comparable by inclusion. The dash-and-dot line represents an edge which is present in one case (red induced $2P_4$) and absent in the other (blue induced $2P_4$) (Color figure online)

#### Lemma 7

For any $i\in \{1,\dotsc ,7\}$, we can reduce *G* to an equivalent set of at most two instances, both of which satisfy $R_i=\emptyset$.

#### Proof

By Lemma 6, we know that any vertex $x\in R_i$ is a full neighbor of each of its adjacent top components.

Let *x*, *y* be two distinct vertices of $R_i$. We claim that the two sets $N_2(x)$ and $N_2(y)$ are comparable by inclusion. To see this, suppose for contradiction that there are vertices $x'\in N_2(x){\setminus } N_2(y)$ and $y'\in N_2(y){\setminus } N_2(x)$. Then we can find in *G* a copy of $2P_4$ in which the first $P_4$ is $v_{i+2}v_{i+3}v_{i+4}v_{i+5}$, and the second $P_4$ is either $x'xyy'$ (if $xy\in E(G)$), or $x'xv_iy$ (if $xy\not \in E(G)$); see Fig. 4.

**Fig. 5:**
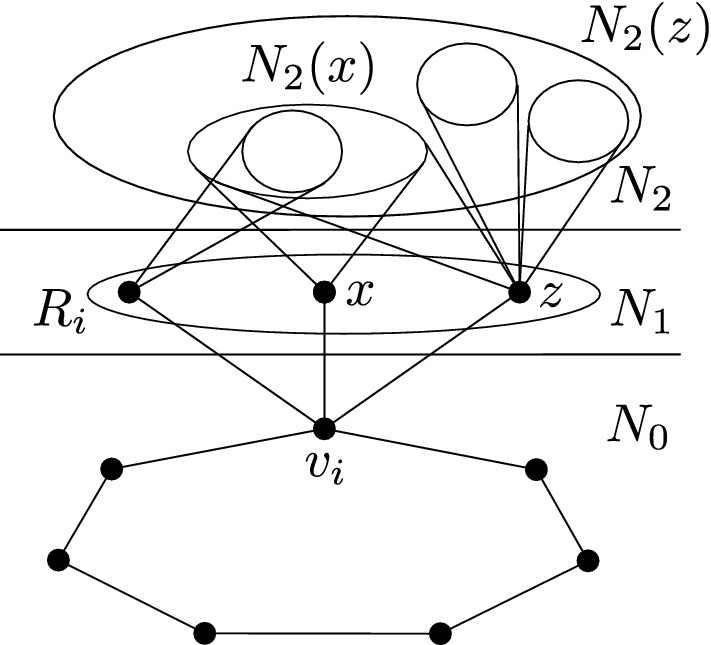
The situation obtained when the neighborhoods of vertices in $R_i$ in $N_2$ are comparable by inclusion with $N_2(z)$ being the largest neighborhood. Note that these vertices are full neighbors of their top components

Choose $z\in R_i$ so that $N_2(z)$ is as large as possible. In particular, for every $x\in R_i$, we have $N_2(x)\subseteq N_2(z)$. We then obtain two instances equivalent to *G* by coloring *z* by its two available colors. Note that by coloring *z*, we ensure that all the vertices $N_2(z)$ have at most two available colors, and since *z* is a full neighbor of all its adjacent top components, this ensures that the vertices of $R_i$ will no longer be relevant after *z* has been colored; see Fig. 5 for illustration. $\square$

From now on, we deal with instances of *G* where every relevant vertex has exactly two neighbors in $N_0$. Let $S_i$ be the set of relevant vertices adjacent to $v_i$.

#### Lemma 8

For any $i\in \{1,\dotsc ,7\}$, we can reduce *G* to an equivalent set of polynomially many instances, each of which satisfies $S_i=\emptyset$ or $S_{i+3}=\emptyset$.

#### Proof

Suppose that the vertices in $S_i$ have available colors 1 and 2, while the vertices in $S_{i+3}$ have available colors 2 and 3 (the case when the vertices in $S_{i+3}$ have the same available colors as the vertices in $S_i$ is similar and we omit it).

For a pair of vertices $x\in S_i$ and $y\in S_{i+3}$, we distinguish the following three possibilities:

($$\alpha $$)
$N_2(x)$ and $N_2(y)$ are comparable by inclusion,
($$\beta $$)
*x* is adjacent to *y*, or
($$\gamma $$)
neither of the previous two conditions holds.

**Fig. 6:**
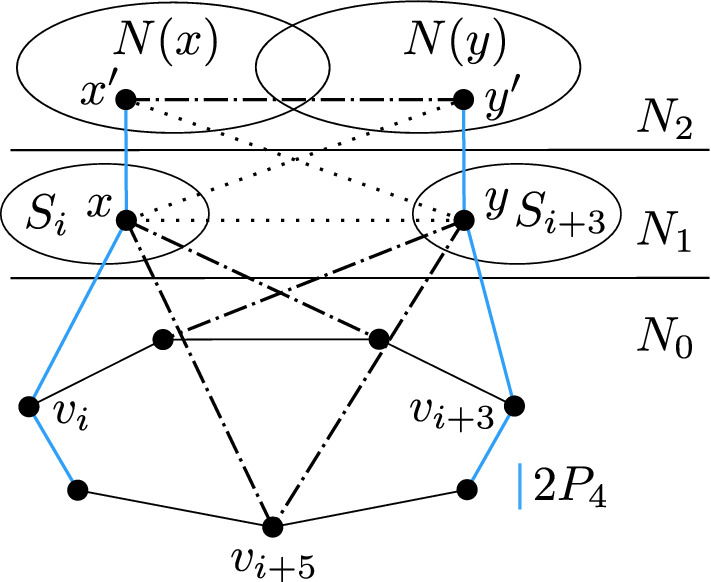
Considering a pair of vertices (*x*, *y*) of type $\gamma$ for $x \in S_i, y \in S_{i+3}$, the edge $x'y'$ must be present, otherwise we obtain an induced $2P_4$. The other dash-and-dotted edges are not necessarily present, and the vertex $v_{i+5}$ is adjacent to at most one vertex from $\{x, y\}$

We say that the pair (*x*, *y*) is of *type* $\alpha$ if it satisfies the condition $(\alpha )$ above, and similarly for the other two types. Observe that if the pair (*x*, *y*) is of type $\gamma$, then there exist $x'\in N_2(x){\setminus } N_2(y)$ and $y'\in N_2(y){\setminus } N_2(x)$. Moreover, for any choice of such $x'$ and $y'$, the pair $x'y'$ must be an edge of *G*, otherwise $x'xv_i v_{i-1}$ and $y'yv_{i+3}v_{i+4}$ would form a copy of $2P_4$. In particular, $x'$ and $y'$ belong to the same top component *C*, and both *x* and *y* are partial neighbors of *C*, as is depicted in Fig. 6.

**Fig. 7:**
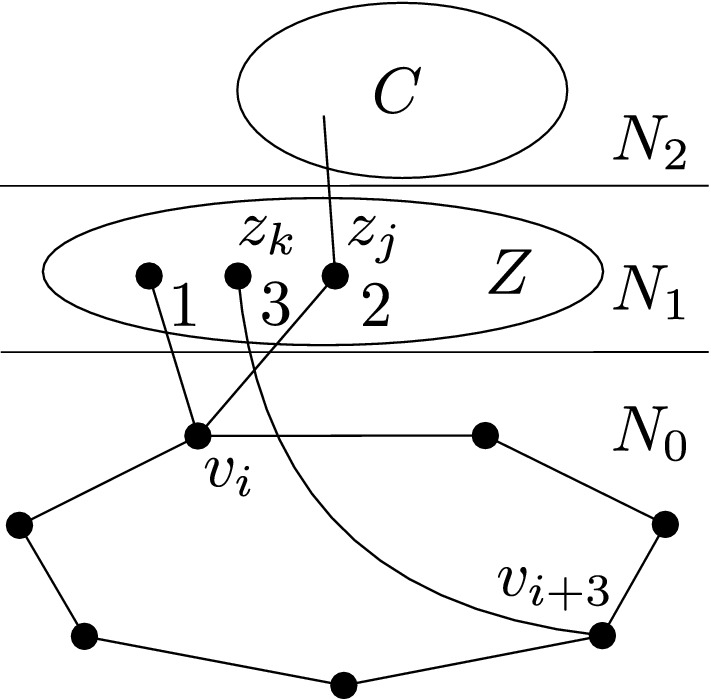
Coloring (*b*) from the proof of Lemma 8 for $k<j$

Let $Z=S_i\cup S_{i+3}$. Let *m* be the size of *Z*, and let us order the vertices of *Z* into a sequence $z_1,z_2,\dotsc ,z_m$ satisfying $|N_2(z_1)|\ge |N_2(z_2)|\ge \cdots \ge |N_2(z_m)|$.

We will reduce *G* to the set of all the instances that can be constructed by one of the following two rules:

- All the vertices in *Z* are colored by their available color different from 2 (i.e., the vertices of $S_i$ are colored by 1, the vertices of $S_{i+3}$ by 3).
- Fix a $j\in \{1,\dotsc ,m\}$ and proceed as follows: color the vertices $z_1,\dotsc ,z_{j-1}$ by their available color different from 2, and color $z_j$ by 2. Moreover, if $z_j$ is a partial neighbor of a top component *C* (note that by Lemma 6, $z_j$ is not adjacent to any other top component), color a dominating set of size two in *C*, in all the possible ways.

We now verify that in all the colorings described above, after all possible basic reductions are applied, either $S_i$ or $S_{i+3}$ becomes empty. This is clearly the case for the coloring described in (a), in which all the vertices in $S_i\cup S_{i+3}$ will be removed from *G*, so both sets will be empty.

Consider now a coloring described in (b), and assume without loss of generality that $z_j$ is in $S_i$. We claim that after the coloring is performed, there will be no relevant vertex left in $S_{i+3}$. To see this, consider a vertex $z_k\in S_{i+3}$. If $k<j$, then $z_k$ has been colored by the color 3, see Fig. 7.

If $k>j$, we distinguish three possibilities depending on the type of the pair $(z_j,z_k)$. If the pair $(z_j,z_k)$ is of type $\alpha$, then $N_2(z_k)\subseteq N_2(z_j)$ (recall that $k>j$ implies $|N_2(z_j)|\ge |N_2(z_k)|$). In particular, all the vertices in any top component adjacent to $z_k$ will only have two available colors (recall that if $z_j$ is a partial neighbor of a top component, we also color a dominating set of this top component, ensuring all its vertices have at most two available colors). Thus, $z_k$ will no longer be relevant. If the pair $(z_j,z_k)$ is of type $\beta$, i.e. $z_j z_k$ is an edge, then $z_k$ has only the color 3 available and can be colored. Finally, suppose $(z_j,z_k)$ is of type $\gamma$. As discussed before, this means both $z_j$ and $z_k$ are partial neighbors of a top component *C* and have no other neighbors in $N_2$. After the coloring is performed, all the vertices in *C* will have only two available colors, because we have colored its dominating set of size two. Hence $z_k$ is no longer relevant. We conclude that $S_{i+3}$ becomes empty, as claimed.

It is clear that the coloring rules (a) and (b) admit only polynomially many possible colorings, and that any valid list coloring of *G* extends one of the partial colorings described in (a) or in (b). Thus, we reduced *G* to an equivalent set of polynomially many instances. $\square$

From now on, assume that we deal with an instance *G* in which for every *i*, one of the two sets $S_i$ and $S_{i+3}$ is empty. Unless the instance is already completely solved, there must be at least one relevant vertex. Assume without loss of generality that *G* has a relevant vertex adjacent to $v_1$ and $v_3$. It follows that $S_1$ and $S_3$ are nonempty, and hence $S_4$, $S_5$, $S_6$ and $S_7$ are empty. Moreover, as any relevant vertex is adjacent to a pair of vertices of the form $\{v_i,v_{i+2}\}$, it follows that $S_2$ is empty as well. In particular, every relevant vertex *x* satisfies $N_0(x)=\{v_1,v_3\}$. It follows that all the relevant vertices have the same palette of size 2; assume without loss of generality that this palette is $\{1,2\}$.

**Fig. 8:**
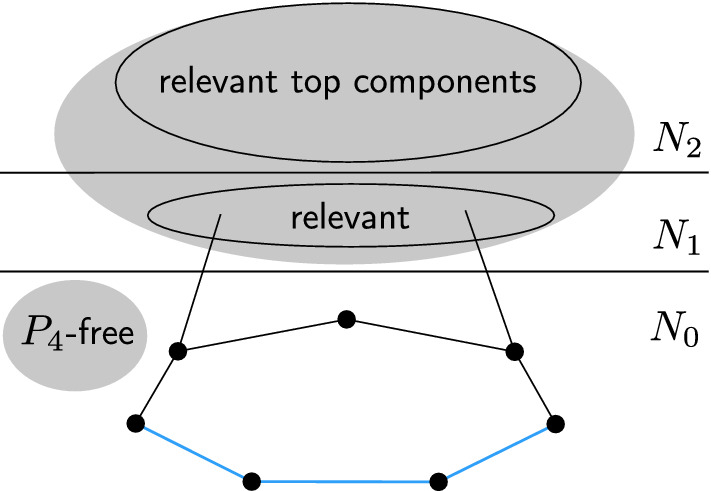
The situation considered in the remaining part of the main proof starting with Lemma 9

We will now focus on describing the structure of the subgraph of *G* induced by the relevant vertices and the relevant top components adjacent to them. Let *R* denote the set of relevant vertices. Note that the subgraph of *G* induced by $R\cup N_2$ does not contain $P_4$, otherwise we could use the path $v_4v_5v_6v_7$ to get a $2P_4$ in *G*; see Fig. 8.

Note also that if two relevant vertices *x* and *y* are adjacent, then any common neighbor of *x* and *y* must be colored by color 3, thanks to the diamond consistency rule. We thus know that adjacent relevant vertices have no common neighbors outside $N_0$. We may also assume that the graph induced by the relevant vertices is bipartite, which we checked as a second step at the beginning of our algorithm. Otherwise, we rejected such an instance as *G* was clearly not 3-colorable.

#### Lemma 9

Suppose that *x* and *y* are two adjacent relevant vertices. Let us write $X'=N_2(x)$ and $Y'=N_2(y)$. Then there are disjoint sets $X, Y\subseteq R$, with $x\in X$ and $y\in Y$, satisfying these properties:

1. Every vertex in $X\cup Y'$ is adjacent to every vertex in $Y\cup X'$.
2. *X* and *Y* are independent sets of *G*.
3. The vertices in $X'\cup Y'$ are only adjacent to vertices in $X\cup Y\cup X'\cup Y'$; in particular, $X'\cup Y'$ induce a top component.

#### Proof

Consider the subgraph *G*[*R*] of *G* induced by the relevant vertices, and let *C* be the connected component of *G*[*R*] containing *x* and *y*. Recall that *C* must be bipartite. We let *X* and *Y* be its partite classes containing *x* and *y*, respectively. Note that *C* is complete bipartite. Otherwise, it would contain a $P_4$.

We will now show that all the vertices in *X* have the same neighbors in $N_2$. Indeed, if we could find a pair of vertices $x_1, x_2\in X$ and a vertex $x'\in N_2(x_1)$ not adjacent to $x_2$, then $x' x_1yx_2$ would induce a $P_4$. It follows that for every $x_1\in X$ we have $N_2(x_1)=X'$, and similarly for every $y_1\in Y$ we have $N_2(y_1)=Y'$.

We saw that adjacent relevant vertices have no common neighbors in $N_2$, so $X'$ and $Y'$ are disjoint. Every vertex in $X'$ must be adjacent to every vertex in $Y'$, for if there were nonadjacent vertices $x'\in X'$ and $y'\in Y'$, then $x'xyy'$ would induce a $P_4$. This proves the first claim of the lemma.

To prove the second claim, observe that *X* and *Y* are independent by construction.

To prove the third claim, proceed by contradiction and assume that a vertex $x'\in X'\cup Y'$ is adjacent to a vertex *z* not belonging to $X\cup Y\cup X'\cup Y'$. We may assume that $x'$ belongs to $X'$. Necessarily, *z* belongs to $R\cup N_2$, and $zx'xy$ induces a forbidden $P_4$. $\square$

Suppose *G*[*R*] contains at least one edge *xy*, and let $X,Y,X',Y'$ be as in the previous lemma. Note that there are only two possible ways to color $G[X\cup Y]$ – either *X* is colored 1 and *Y* is colored 2, or vice versa. We can check in polynomial time which of these two colorings can be extended to a valid coloring of $G[X\cup Y\cup X'\cup Y']$. If neither of the two colorings extends, we reject the list 3-coloring instance, if only one of the two colorings extends, we color $X\cup Y$ accordingly, and if both colorings extend, we remove the vertices $X'\cup Y'$ from *G*, resulting in a smaller equivalent instance, in which $X\cup Y$ is no longer relevant. Repeating this for every component of *G*[*R*] that contains at least one edge, we eventually reduce the problem to an instance in which the relevant vertices form an independent set.

From now on, we assume *R* is independent in *G*. For a vertex $x\in R$, let $C_2(x)$ denote the set of top components that contain at least one neighbor of *x*.

#### Lemma 10

For any two relevant vertices *x* and *y*, we either have $C_2(x)=C_2(y)$, or $C_2(x)$ and $C_2(y)$ are disjoint.

#### Proof

Suppose the lemma fails for some *x* and *y*. We may then assume that there is a top component $C\in C_2(x)\cap C_2(y)$ and a component $C'\in C_2(x){\setminus } C_2(y)$. Since $|C_2(x)|\ge 2$, we know from Lemma 6 that *x* is a full neighbor of all the top components in $C_2(x)$. Choose a vertex $u\in C'$ and a vertex $v\in C\cap N_2(y)$. Then *uxvy* is a copy of $P_4$ in $R\cup N_2$, which is impossible. $\square$

Let us say that two relevant vertices *x* and *y* are *equivalent* if $C_2(x)=C_2(y)$. As the next step in our algorithm, we will process the equivalence classes one by one, with the aim to reduce the instance *G* to an equivalent instance in which each relevant vertex is adjacent to a single top component.

Let $x\in R$ be a vertex such that $|C_2(x)|\ge 2$, and let $R_x$ be the equivalence class containing *x*. By Lemma 6, each vertex in $R_x$ is a full neighbor of any component in $C_2(x)$, and by Lemma 10, no vertex outside of $R_x$ may be adjacent to a relevant top component in $C_2(x)$. Thus, $R_x$ is a vertex cut separating the relevant top components in $C_2(x)$ from the rest of *G*. We may therefore apply the cut reduction through the vertex cut $R_x$ to reduce *G* to a smaller instance in which the vertices of $R_x$ are no longer relevant.

We repeat the cut reductions until there is no relevant vertex adjacent to more than one top component. From now on, we deal with instances in which each relevant vertex is adjacent to a unique top component; note that this top component is necessarily relevant.

#### Lemma 11

Let *x* be a relevant vertex, let *C* be the top component adjacent to *x*, let $R_x$ be the equivalence class of *x*, and let $y\in R_x\cup C$ be a vertex not adjacent to *x*. Then *y* is adjacent to at least one vertex in $N_2(x)$. Moreover, if $N_2(x)$ induces a disconnected subgraph of *G*, then *y* is adjacent to all the vertices of $N_2(x)$.

#### Proof

**Fig. 9:**
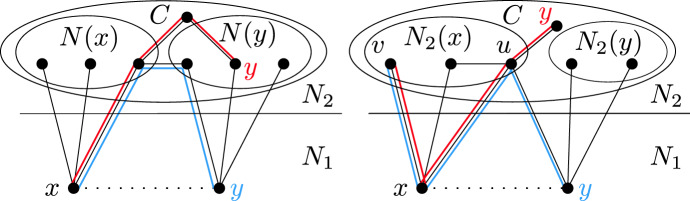
Illustrations to the proof of Lemma 11. The left part shows a situation when *y* is not adjacent to any vertex in $N_2(x)$, the right part shows a situation when *y* has a neighbor in $N_2(x)$ which is disconnected. Each part depicts two different possibilities. The blue $P_4$ shows the case $y \in R_x$, while the red $P_4$ shows the case when $y\in C$ (Color figure online)

Refer to Fig. 9. If *y* is not adjacent to any vertex of $N_2(x)$, then we can find an induced path with at least four vertices by considering the shortest path from *x* to *y* in the graph induced by $C\cup \{x,y\}$. Therefore *y* has at least one neighbor in $N_2(x)$. Suppose now that $N_2(x)$ is disconnected. If *y* is not adjacent to all the vertices of $N_2(x)$, then we can find a vertex $u\in N_2(x)$ adjacent to *y*, and a vertex $v\in N_2(x)$ nonadjacent to *y*, in such a way that *u* and *v* are in distinct components of $N_2(x)$. Then *yuxv* is an induced $P_4$. $\square$

Fix now a relevant top component *C* and let *R* be set of relevant vertices in $N_1$ adjacent to *C*. Fix a vertex $x\in R$ so that $N_2(x)$ is as large as possible. Let $R_x$ be the equivalence class containing *x*. We distinguish several possibilities, based on the structure of $N_2(x)$.

#### $N_2(x)$ is Disconnected

Suppose first that $N_2(x)$ induces in *G* a disconnected subgraph. By Lemma 11, any vertex in $R_x$ is adjacent to all vertices in $N_2(x)$. By our choice of *x*, this implies that for any $x'\in R_x$ we have $N_2(x')=N_2(x)$. We may therefore apply the cut reduction for the cut $R_x$ that separates *C* from the rest of *G*, to obtain a smaller instance in which the vertices of $R_x$ are no longer relevant.

#### $N_2(x)$ is Connected, with $\ge 3$ Vertices

**Fig. 10:**
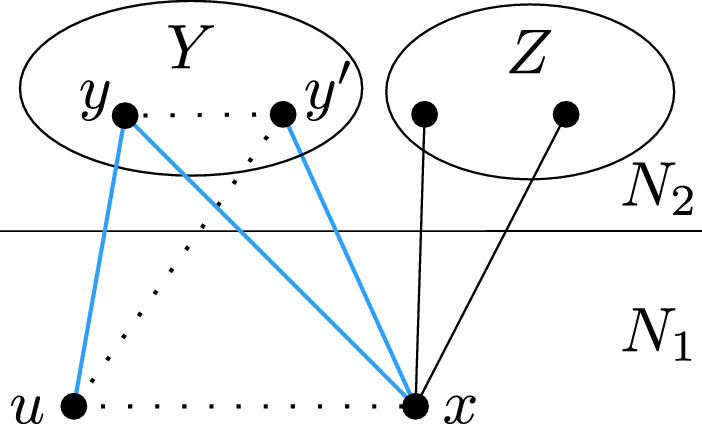
There is an induced $P_4$ in $N_2$ if $N_2(x)$ is connected with $\ge 3$ vertices and for $y, y' \in Y$ there exists a *u* neighboring only one of them

Now suppose that $N_2(x)$ induces a connected graph, and that $N_2(x)$ has at least three vertices. We now verify that $N_2(x)$ induces a complete bipartite graph, otherwise *C* contains $P_4$ or *G* is not 3-colorable. Let *Y* and *Z* be the two partite classes of $N_2(x)$. Note that any two vertices $y,y'$ in *Y* have the same neighbors in *G*: indeed if *u* were a vertex adjacent to *y* but not to $y'$, then $uyxy'$ would induce a copy of $P_4$, as depicted in Fig. 10. By the same argument, all the vertices in *Z* have the same neighbors in *G* as well. Diamond consistency enforces that all the vertices in *Y* have the same palette, and similarly for *Z*. We may then invoke neighborhood domination to delete from *Y* all vertices except a single vertex *y*, and do the same with *Z*, reducing *G* to an equivalent instance in which $N_2(x)$ consists of a single edge.

#### $N_2(x)$ is a Single Vertex

Suppose that $N_2(x)$ consists of a single vertex *y*. If *y* is the only vertex of *C*, then *y* must have the palette $\{1,2,3\}$, otherwise *C* would not be a relevant component. In such case, we may simply color *y* with color 3 and delete it, as this does not restrict the possible colorings of $G-y$ in any way. If, on the other hand, *C* has more than one vertex, it follows from Lemma 11 that all the vertices of $R_x$ are adjacent to *y*, and by the choice of *x*, every vertex in $R_x$ is adjacent to *y* as its only neighbor in *C*. We may then apply cut reduction for the cut $R_x$. In all cases, we obtain a smaller equivalent instance, in which the vertices in $R_x$ are no longer relevant.

#### $N_2(x)$ is a Single Edge

The last case to consider deals with the situation when $N_2(x)$ contains exactly two adjacent vertices *u* and *v*. Assume that $\deg _G(u)\ge \deg _G(v)$. Recall that the set *R* of relevant vertices is independent. Note that for any vertex $x'\in R_x$, $N_2(x')$ is connected, otherwise Lemma 11 implies that $N_2(x')$ is contained in $N_2(x)$, contradicting $N_2(x)$ being a single edge.

**Fig. 11:**
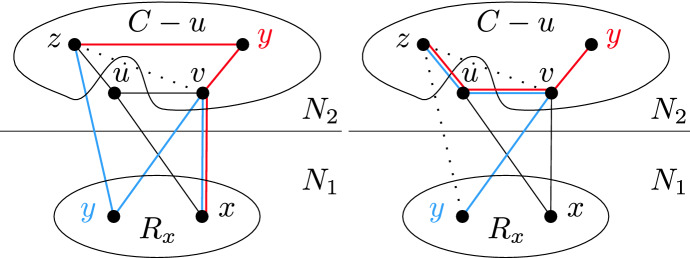
Illustrations of the situation when $N_2(x)$ is a single edge *uv*. Again, case $y \in R_x$ is shown as blue *y* and blue $P_4$, while $y \in (C-u)$ is shown as red *y* and red $P_4$. There are two subcases corresponding to *yz* being an edge or not (Color figure online)

We first claim that any vertex $y\in R_x\cup (C-u)$ adjacent to *v* is also adjacent to *u*. Suppose this is not the case. Then, since $\deg _G(u)\ge \deg _G(v)$, there must also be a vertex $z\in R_x\cup (C-v)$ adjacent to *u* but not to *v*. If *yz* is an edge, then *zyvx* is a copy of $P_4$, and if *yz* is not an edge, then *zuvy* is a copy of $P_4$, as shown in Fig. 11. In both cases we have a contradiction, establishing the claim. Note that the claim, together with Lemma 11, implies that *u* is adjacent to all the other vertices of $R_x\cup C$.

**Fig. 12:**
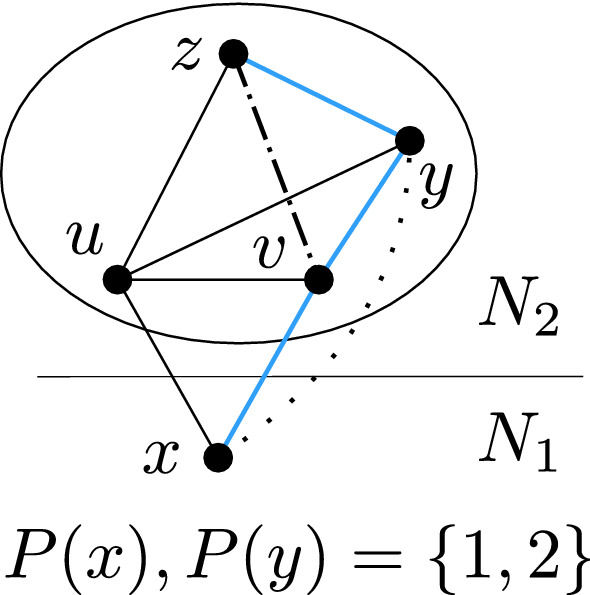
Recall that *u* is adjacent to all vertices of *C*. If there is a $y \in C$ adjacent to both *u* and *v*, there is no other neighbor *z* of *y*. Otherwise, either *zv* is an edge, causing a $K_4$, or *zv* is not an edge, causing an induced $P_4$

Next, we show that if *C* contains a vertex adjacent to both *u* and *v*, then we may reduce *G* to a smaller equivalent instance. Suppose $y\in C$ is adjacent to *u* and *v*. Then $P(y)=P(x)=\{1,2\}$ by diamond consistency. We now claim that *y* has no other neighbors in *G* beyond *u* and *v*. Suppose that $z\not \in \{u,v\}$ is a neighbor of *y*. Then *z* cannot be adjacent to *v*, since *uvyz* would form a $K_4$. Therefore *zyvx* is a copy of $P_4$, a contradiction illustrated by Fig. 12. We conclude that $N(y)=\{u,v\}\subseteq N(x)$, and since $P(y)=P(x)$, we may delete *y* due to neighborhood domination.

From now on, we assume that *u* and *v* have no common neighbor in *C*. Recall that *u* is adjacent to all the other vertices in $C\cup R_x$. We now reduce *G* to an instance where $C-u$ is an independent set. We already know that *v* is isolated in $C-u$ by the previous paragraph. Suppose that $C-u$ has a component *D* with more than one vertex. Suppose *D* has a vertex $v'$ adjacent to a vertex $x'\in R_x$. Let $y'$ be any vertex in $N(v')\cap D$. Observe that $x'y'$ is not an edge as otherwise $x'y'v'u$ is a $K_4$. Hence, $x'y'v'u$ form a diamond. Then $P(y')=P(x')$ by diamond consistency. We claim that $y'$ has no other neighbors in *G* beyond *u* and $v'$. Suppose that $z'\not \in \{u,v'\}$ is a neighbor of $y'$. Then $z'$ cannot be adjacent to $v'$, since $uv'y'z'$ would form a $K_4$. If $z'x'$ is not an edge $z'y'v'x'$ is a copy of $P_4$, a contradiction. If $z'x'$ is an edge then all $z'v'u$ are in $N_2(x)$ contradicting the choice of *x* so that $N_2(x)$ is the largest possible.

We can repeat the reasoning of the previous paragraph with $x'$ and $v'$ in the place of *x* and *v*, showing that *u* and $v'$ cannot have any common neighbor in *C*, contradicting the assumption that *D* has more than one vertex. We can thus conclude that *D* is not adjacent to any vertex in $R_x$. Then *u* is a cut-vertex separating *D* from the rest of *G*. We may test which colorings of *u* can be extended into *D* (since *D* is $P_4$-free, this can be done efficiently), then restrict the palette of *u* to only the feasible colors, and then delete *D*.

**Fig. 13:**
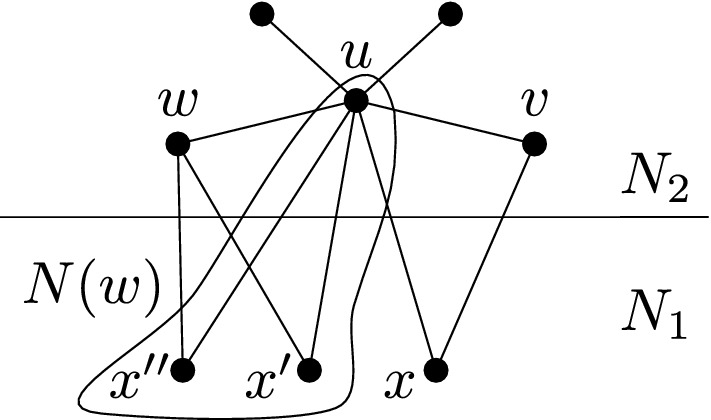
Situation in which we can apply a neighborhood collapse

We are now left with a situation when *C* is a star with center *u*, and every vertex of $R_x$ is adjacent to *u* and to at most one vertex of $C-u$. If there is a vertex $w\in C-u$ adjacent to more than one vertex in $R_x$, it means that the neighborhood of *w* is a connected bipartite graph (a star with center *u*) to which we may apply neighborhood collapse; see Fig. 13.

Suppose now that every vertex $w\in C-u$ has only one neighbor in $R_x$ (if *w* had no neighbor in $R_x$, it would have degree 1 and we could remove it). If *w*’s palette has 3 colors, we can remove it, so we may assume that every vertex in $C-u$ has a palette of size 2. Then *u*’s palette must have 3 colors. Otherwise, *C* would not be a relevant component. If a vertex in $C-u$ has palette $\{1,2\}$, then *u* must be colored 3 and then $R_x$ is no longer relevant.

**Fig. 14:**
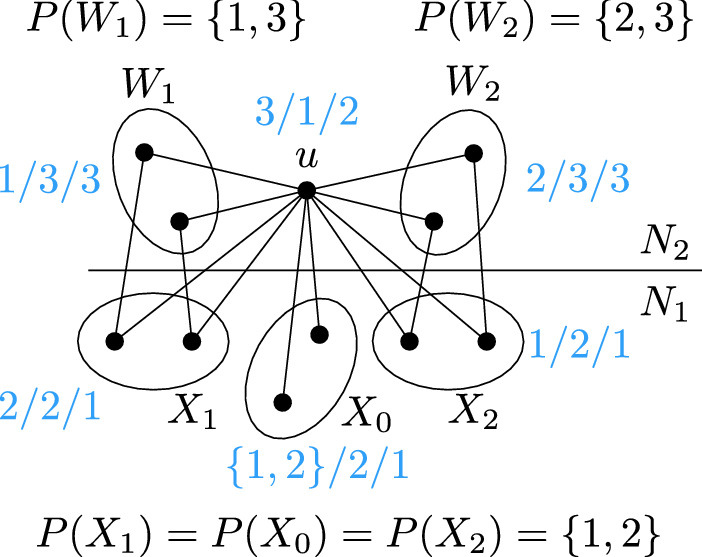
The last case in which each vertex of $C-u$ has palette $\{1,3\}$ or $\{2, 3\}$. The blue text represents the three possibilities to color *u* and what colors that implies for other parts of the graph

It remains to consider the case when each vertex of $C-u$ has the palette $\{1,3\}$ or $\{2,3\}$. Let $W_1$ and $W_2$ be the sets of vertices of $C-u$ having palette $\{1,3\}$ and $\{2,3\}$, respectively. Let $X_1$ and $X_2$ be the sets of vertices of $R_x$ that are adjacent to a vertex in $W_1$ and $W_2$, respectively. Let $X_0$ be the set of vertices in $R_x$ that have no neighbor in $C-u$. The situation is shown in Fig. 14. Let us consider the possible colorings of $C\cup R_x$. If *u* is colored by 3, then the whole set $W_1$ is colored by 1, $W_2$ is colored by 2, hence $X_1$ is colored by 2 and $X_2$ by 1, while the vertices in $X_0$ can be colored arbitrarily by 1 or 2. On the other hand, if *u* receives a color $\alpha \ne 3$, then all the vertices in $R_x$ receive the color $\beta \in \{1,2\}{\setminus }\{\alpha \}$, and the vertices in $C-u$ can be colored by 3. The set $R_x$ therefore admits three types of feasible colorings: the all-1 coloring, the all-2 coloring, and any coloring where the set $X_1$ is colored by 2 and $X_2$ by 1. This set of colorings can be equivalently characterized by the following properties:

- If a vertex in $X_1$ is colored by 1, then the whole $R_x$ receives 1.
- If a vertex in $X_2$ is colored by 2, then the whole $R_x$ is colored by 2.
- All the colors in $X_1$ are equal and all the colors in $X_2$ are equal.

The above properties can be encoded by a 2-SAT formula whose variables correspond to vertices of $R_x$.

To summarize, we have shown that a 3-coloring instance *G* can be reduced to an equivalent set of polynomially many simpler list-3-coloring instances. The structure of these simpler instances guarantees that for any relevant top component *C*, we can form a 2-SAT formula describing the colorings of the relevant vertices adjacent to *C* that can be extended to a proper coloring of *C*. Moreover, in the subgraph of *G* induced by the vertices not belonging to any relevant top component, each vertex has a palette of size at most two. The colorings of this subgraph can again be encoded by a 2-SAT formula. Such an instance of list-3-coloring then admits a solution if and only if there is a satisfying assignment for the conjunction of the 2-SAT formulas described above. The existence of such an assignment can be found in polynomial time. This completes the proof of Theorem 1.

## Conclusions

We have shown that 3-coloring on $(2P_4,C_5)$-free graphs is solvable in polynomial time. As we discussed in the introduction, this approach might serve as a step towards resolving 3-coloring on $2P_4$-free graphs because it remains to consider $2P_4$-free graphs containing $C_5$.

Apart from the main question above, under more refined scale, the complexity of 3-coloring on $(2P_4,C_3)$-free, $(P_8,C_3)$-free, or $(P_8,C_5)$-free graphs remains unknown. In another direction, it would be interesting to extend our result to the list 3-coloring setting.

## Footnotes

1 First two cases were explicitly mentioned as open in [26, 27], the latter two cases are open to the best of our knowledge.

## Declarations

### Editorial Policies for

Springer journals and proceedings: https://www.springer.com/gp/editorial-policies

### Nature Portfolio journals:

https://www.nature.com/nature-research/editorial-policies

### Scientific Reports

https://www.nature.com/srep/journal-policies/editorial-policies

### BMC journals:

https://www.biomedcentral.com/getpublished/editorial-policies
